# Prenatal exposure to paracetamol and risk of autism spectrum disorder: systematic review and meta-analysis of observational studies

**DOI:** 10.61622/rbgo/2026rbgo108

**Published:** 2026-05-12

**Authors:** Juliana Almeida Oliveira, Maria Julia Lemos, Laura Fonseca Queiroz, João Vitor Sciorilli, Tam Tran, Isabella de Oliveira Ortiz, Bruna Benigna Sales Armstrong, Flávia Ribeiro de Oliveira

**Affiliations:** 1 Universidade Federal de Minas Gerais Department of Surgery Belo Horizonte MG Brazil Department of Surgery, Universidade Federal de Minas Gerais, Belo Horizonte, MG, Brazil.; 2 Centro Universitário Redentor Itaperuna RJ Brazil Centro Universitário Redentor, Itaperuna, RJ, Brazil.; 3 Santa Casa de Misericórdia de Belo Horizonte Department of Obstetrics and Gynecology Belo Horizonte MG Brazil Department of Obstetrics and Gynecology, Santa Casa de Misericórdia de Belo Horizonte, Belo Horizonte, MG, Brazil.; 4 Centro Universitário Faculdade de Medicina do ABC Santo André SP Brazil Centro Universitário Faculdade de Medicina do ABC, Santo André, SP, Brazil.; 5 Washington University School of Medicine Saint Louis MO United States of America Washington University School of Medicine, Saint Louis, MO, United States of America.; 6 Hospital Santa Marcelina São Paulo SP Brazil Hospital Santa Marcelina, São Paulo, SP, Brazil.; 7 Irmandade Santa Casa de Misericórdia São Paulo SP Brazil Irmandade Santa Casa de Misericórdia, São Paulo, SP, Brazil.; 8 Universidade Federal de Minas Gerais Department of Obstetrics and Gynecology Belo Horizonte MG Brazil Department of Obstetrics and Gynecology, Universidade Federal de Minas Gerais, Belo Horizonte, MG, Brazil.

**Keywords:** Acetaminophen, Paracetamol, Pregnancy, Autism spectrum disorder

## Abstract

**Objective:**

This meta-analysis aimed to assess the ASD risk in offspring exposed to prenatal acetaminophen compared to non-exposed offspring.

**Methods:**

A systematic search was conducted across PubMed, Embase, and Cochrane Central Register of Controlled Trials databases up to October 2025. Studies were included if they involved pregnant women, compared exposed versus non-exposed groups, were RCTs or cohort studies, and reported ASD outcomes. Data was extracted independently by two authors, with discrepancies resolved by consensus. Statistical analyses used odds ratios (ORs) with 95% confidence intervals, Cochran Q, and I² statistics with a random-effects model. Study quality was appraised using the ROBINS-E tool.

**Results:**

Eight studies, involving 2,560,208 patients, were included. Pooled results showed an 18% increased risk of ASD diagnosis (p <0.0001) and a non-significant 16% increase for ASD symptoms (p=0.1719). Dose-response relationships and gender-specific effects were reported by studies, while familial confounding and "some concerns" to "high" risk of bias were identified.

**Conclusion:**

A consistent, albeit modest, association was found. These findings emphasize the necessity for careful benefit-risk assessments and informed dialogue with expectant mothers regarding pain and fever management during pregnancy.

**PROSPERO**: CRD420251160888

## Introduction

Acetaminophen is widely accepted as the first-line over-the-counter analgesic and antipyretic in pregnancy, given its favorable safety profile compared to nonsteroidal anti-inflammatory drugs or other analgesics.^(1)^ For this, it is estimated that over 50% of pregnant women used acetaminophen at some point during pregnancy,^(1,2)^ despite the reluctance of using medication during pregnancy. Approximately, eight out of 10 women take at least one or over-the-counter medication during gestation.^(3,4)^

In the past years, a growing amount of evidence has raised concerns that acetaminophen exposure might be associated with elevated risks of neurodevelopmental disorders, including autism spectrum disorder (ASD) and attention-deficit/hyperactivity disorder (ADHD).^(5-8)^ A recent systematic review including 46 epidemiologic studies found that the majority reported positive associations between prenatal acetaminophen use and subsequent neurodevelopmental outcomes in offspring.^(1)^

However, evidence remains controversial. A large cohort failed to identify significant associations, while sibling-comparison and negative control analyses have questioned whether residual confounding or underlying maternal indications might explain observed associations.^(7)^

Recent discussions on the pros and cons of acetaminophen use and autism rates, raised medical societies and health entities to advocate on the topic. Recently, the U.S. Food and Drug Administration (FDA) notified physicians to minimize acetaminophen prescription during pregnancy, due to these concerns.^(9)^ Additionally, the American College of Obstetricians and Gynecologists (ACOG) highlights that acetaminophen is safe for use during pregnancy when taken appropriately, underscoring the necessity of a careful benefit-risk assessment.^(2)^ Also, the Society for Maternal-Fetal Medicine (SMFM) highlighted that the risks associated with untreated maternal fever or pain may outweigh those linked to the responsible use of acetaminophen, especially due to the lack of causal relationship^(10)^ and of conclusive evidence of its associating with autism.^(11)^

Previous reviews have pointed to heterogeneity in exposure definitions, confounding control, and outcome ascertainment as key limitations.^(1,12)^ Thus, the present meta-analysis aims to assess ASD risk in pregnant women offspring who were exposed *versus* not-exposed to acetaminophen.

## Methods

### Registration

This systematic review and meta-analysis was guided by the principles outlined in the Cochrane Handbook for Systematic Reviews of Interventions^(13)^ and the recommendations of the Preferred Reporting Items for Systematic Reviews and Meta-Analysis (PRISMA) statement.^(14)^ The protocol was registered with the International Prospective Register of Systematic Reviews (PROSPERO) in October 2025, under the identification number CRD420251160888.

### Studies eligibility, information sources and search strategy

Studies were considered eligible if they satisfied all of the following conditions: (1) inclusion of pregnant women; (2) exposure *versus* non-exposure comparison; (3) randomized controlled trials (RCTs) and cohort studies; and (4) presented any outcome associated with the ASD. Studies were excluded if they lacked (1) a Pacontrol group; (2) sufficient follow-up data; or were published as (3) conference abstracts, (4) letters, or (5) editorials. No restrictions were applied regarding the language of publication or the duration of the follow-up period.

A systematic search was conducted across the PubMed, Embase, and Cochrane Central Register of Controlled Trials databases, covering publications from their inception up to October 2025. The search utilized a combination of the following keywords and their relevant synonyms: "acetaminophen", "autism spectrum disorder", "asperger syndrome", "autistic disorder". We did not limit the search string to outcomes. The complete search strategy is available in the Supplemental Material: Table S1. Our search methodology did not incorporate any filters for language or publication type, specifically excluding grey literature.

### Selection process, data items and data collection process

We assessed all outcomes available, considering this might be the biggest poll of patients assessing autism related outcomes. However, due to the small number of studies, we were only able to assess the outcomes of Autism Spectrum Disorder (ASD) diagnosis and ASD symptoms presence. Four studies reported the outcome as presence of ASD symptoms^(5,15-17)^ and four as diagnosis of ASD.^(7,18-20)^ Considering the previous systematic reviews approach,^(12)^ we also did a pooled analysis of both outcomes together. This strategy enabled funnel plot analysis, since a minimum or 8 to 10 studies is necessary for publication bias assessment.

Due to lack of data, we were unable to perform statistical evaluations for subgroups. such as treatment dose, duration and frequency, among others.

All retrieved studies were managed and uploaded onto the Rayyan platform.^(21)^ A meticulous manual review of the reference lists from all included studies was performed to identify any further pertinent research. Data extraction were independently performed by two authors each. Discrepancies that arose from independent evaluations were resolved through mutual consensus between the researchers or assessment of another author. For data originally reported as medians and interquartile ranges, conversion to means and standard deviations was executed using the established methodologies described by Luo et al.^(22)^ Authors were contact for full retrieval of papers.

### Synthesis methods

The statistical analysis adhered to the guidelines provided by the Cochrane Collaboration and the PRISMA statement.^(13,14)^ Odds ratios (ORs) with corresponding 95% confidence intervals were employed to compare treatment effects for all categorical endpoints. Heterogeneity across studies was evaluated using the Cochran Q test and I² statistics. Substantial heterogeneity was defined by P values less than 0.10 or an I² value greater than 25%. The Restricted Maximum Likelihood random-effect was applied independently of heterogeneity level. Sensitivity analyses were conducted using a leave-one-out strategy. Statistical computations were performed using R statistical software version 4.5.1 (R Foundation for Statistical Computing).

There were two papers that fitted inclusion criteria,^(5,15,16)^ but did not share data in the original publication. However, the authors of the original studies were authors in a previous systematic review,^(12)^ where the data was shared and collected for use in this meta-analysis. The authors of the original studies were contacted, with no response until the date of publication of this meta-analysis.

### Reporting bias

Included studies quality was appraised using the Cochrane Risk-of-Bias tool for Non-randomized Studies of Exposure (ROBINS-E).^(23)^ Two authors independently completed the risk of bias assessment. Any disparities in their evaluations were resolved through discussion and consensus among the authors. The Risk-of-Bias VISualization tool (RoBvis) was used for plot generation.^(24)^

## Results

### Study selection

**Figure 1:**
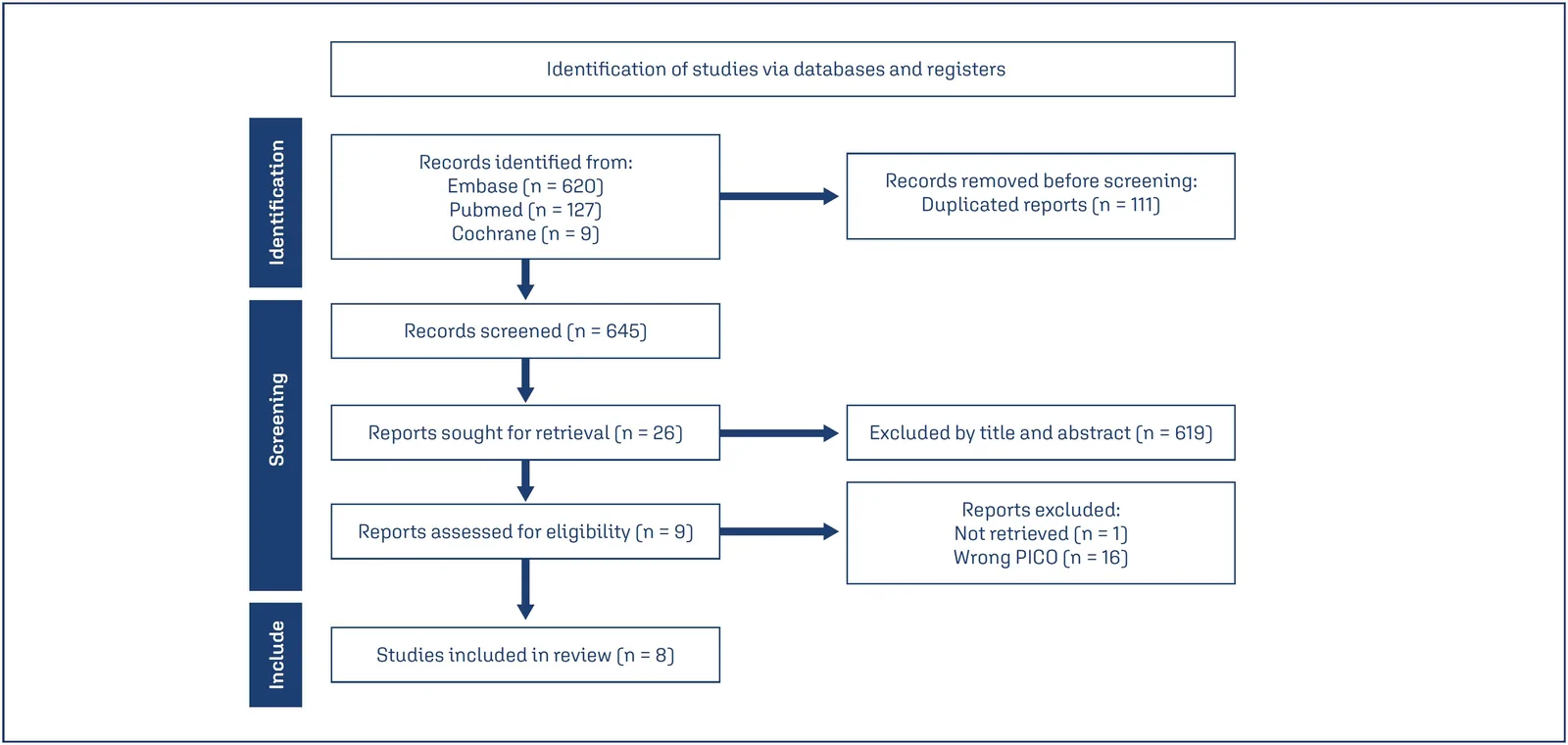
Studies screening PRISMA flow-chart

Of 756 studies, a total of eight studies ^(5,7,15-20)^ (Figure 1), involving 2,560,208 patients were included, of which only 228,115 (8.9%) were exposed to acetaminophen.

### Overall characteristics of the included studies

Reason for acetaminophen use was reported by 3 studies, including: self-reported use for general pain or fever;^(20)^ unspecified pain, infection, among others;^(17)^ and, reporting rates, fever (2.7%), migraine (14.9%), pain (12.4%), infection (11.1%), rheumatoid arthritis (33.3%), asthma (12.7%), and headache (17.8%).^(7)^ Autism assessment was performed at different moments, being cited as the mean age of 14.84 months,^(17)^ 18 months (Modified Checklist for Autism in Toddlers - M-CHAT),^(16)^ and an undetailed range of 9 months to 6.5 years,^(12,16)^ and at any time of life.^(7)^ Only one study cited medication dose, which varied from below 166mg to over 430mg per day.^(7)^ As for frequency, it was related as sporadic (any dose in one or two trimesters; 42.3% out of 1255 participants) and persistent use (any dose in all three trimesters; 3.8%) by one study.^(17)^ All studies were conducted in different countries, being 7 in Europe and 1 in North America (Chart 1).

**Chart 1:** Baseline characteristics of the included studies

Study	Country	Study Type	Sample Size (n)	Follow-up, Years (Mean)	Exposure Type	Maternal Age, Years (Mean ± SD)	Timing of Exposure	Smokers during pregnancy (n)
Ahlqvist et al. (2024)^(7)^	Sweden	National cohort	E: 185,909 NE: 2,294,888	26	Acetaminophen	E: 30.73 ± 5.27 NE: 30.59 ± 5.17	Pregnancy	E: 21,266 NE: 189,115
Avella-García et al. (2016)^(17)^	Spain	Prospective cohort	E: 955 NE: 1240	5	Acetaminophen	31^a^	Pregnancy (assessed at 18 and 32 weeks of GA)	E: 348 NE: 318
Chatzi et al. (2017)^(16)^	Greece	Prospective cohort	345	6	Acetaminophen	30.1 ± 4.2	Pregnancy (at 12 and 30 weeks of GA)	29.1% of total cohort
Ji et al. (2020)^(19)^	USA	Prospective cohort	E: 649 NE: 531	20	Acetaminophen	31.6 ± 3.0^b^	Pregnancy	113
Leppert et al. (2019)^(20)^	UK	Genetic observational	E: 4,415 NE: 3,617	0.4	Acetaminophen	28.5 ± 4.8	Pregnancy	1157
Liew et al. (2016)^(18)^	Denmark	Prospective cohort	E: 36,187 NE: 28,135	12.5	Acetaminophen	E: 29.6 ± 4.1 NE: 29.6 ± 4.0	All trimesters assessed	E: 36,187^c^ NE: 28,135
Porta and Fantini (2006)^(15)^	Italy	Prospective cohort	153	7	Acetaminophen	36.6 ± 6.1	All trimesters assessed	9
Snijder et al. (2012)^(5)^	Netherlands	Prospective cohort	3,184	2.5	Paracetamol, NSAIDs, aspirin	30.29 (5.12)^b^	Periconception and pregnancy (0 to 32 weeks of GA)	9

a SD not reported;

b Maternal age was reported in categorical groups (<25, 25–29, 30–34, 35–39, ≥40 years). To estimate mean and SD, each category was assigned a midpoint (22, 27, 32, 37, and 42 years, respectively). Weighted means and standard deviations were then calculated using group counts;

c Status was collapsed into a binary variable (any smoking vs. never). E: Exposure; GA: Gestational age; N: Number; NE: Not exposed; SD: Standard Deviation; UK: United Kingdom; USA: United States of America

### Pooled Analysis of Included Studies

**Figure 2:**
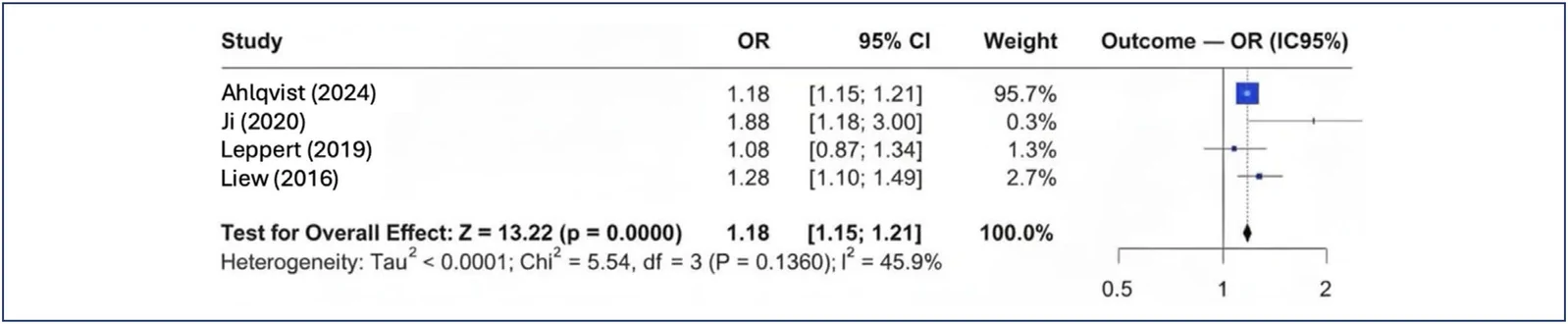
Acetaminophen exposure increased the odds of the offspring presenting an ASD diagnosis by 18%

**Figure 3:**
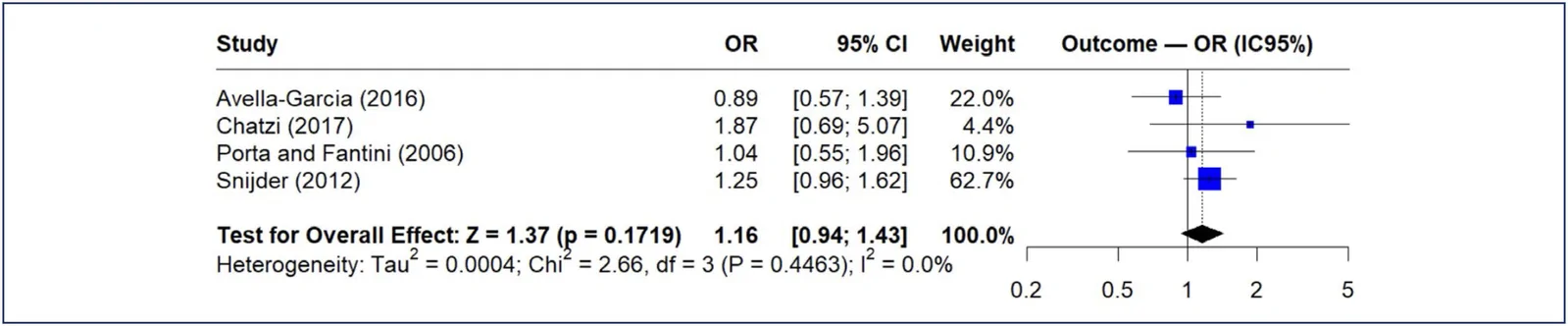
Acetaminophen use led to a non-significant 16% increase in ASD symptoms identification

The pooled results showed that acetaminophen use during pregnancy increased the risk of ASD diagnosis by 18% (OR 1.18, 95% CI 1.15 to 1.32, p <0.0001; I^2^ = 45.9%) (Figure 2). Despite one study increasing overall heterogeneity,^(19)^ the findings still were statistically significant in the leave-one-out analysis (Supplemental material: Figure S1). A higher risk of presenting ASD symptoms by 16% was observed, albeit not statistically significant (OR 1.16, 95% CI 0.94 to 1.43, p= 0.1719, I^2^ = 0%) (Figure 3) and with no substantial heterogeneity in the sensitivity analysis (Supplemental material: Figure S2).

When the outcomes were combined into one, similarly to the strategy from a previous pooled analysis (12), we were able of seeing an overall increase of 18% in ASD diagnosis and symptoms presentations (OR 1.18; 95% CI: 1.15 to 1.21, p <0.0001; I^2^ = 15%; Supp. mat.: Figure S4), with low heterogeneity (Supp. mat.: Figure S5). A funnel plot analysis was performed for overall ASD identification (through diagnosis and symptoms), identifying no publication bias (Supp. mat.: Figure S6).

### Autism Spectrum Disorder (ASD) and Autism Spectrum Symptoms

Multiple studies investigated the link between prenatal acetaminophen exposure and ASD outcomes finding a link to acetaminophen use during pregnancy and autism.^(7,12,15-19)^ Utilizing cord plasma biomarkers, one study reported a dose-dependent increase in ASD diagnosis odds; the odds were 3.62 times higher (95% CI 1.62 to 8.60) for those in the third tertile of cord acetaminophen burden, and 2.29 times higher (95% CI 1.06 to 4.85) with the detection of cord acetaminophen glucuronide.^(19)^ Furthermore, one study identified an overall increased risk for ASD (HR 1.19, 95% CI 1.04 to 1.35) and infantile autism (HR 1.10, 95% CI 0.89 to 1.37) with ever-use of acetaminophen.^(18)^ This risk was more pronounced for ASD co-occurring with hyperkinetic symptoms (HR 1.51, 95% CI 1.19 to 1.92) and infantile autism with hyperkinetic symptoms (HR 1.55, 95% CI 0.98 to 2.45), with no significant association for ASD or infantile autism outcomes when hyperkinetic symptoms were absent.^(18)^ A dose-response relationship was also noted when acetaminophen was used for over 20 weeks, nearly doubling the risk of ASD with hyperkinetic symptoms (HR 1.89, 95% CI 1.19 to 3.02).^(18)^

Gender-specific effects on autism spectrum symptoms (CAST scores) was observed in males exposed to acetaminophen, whom showed an increase in symptom scores (coefficient b = 0.63, 95% CI 0.09 to 1.18), with persistent exposure leading to more pronounced symptoms (coefficient b = 1.91, 95% CI 0.44 to 3.38). Conversely, exposed females displayed a statistically significant decrease in these symptoms (coefficient b = −0.51, 95% CI −0.98 to −0.05).^(17)^ In contrast, while reporting a marginal increase in autism risk in population-based models (HR 1.05, 95% CI 1.02 to 1.08), one study concluded that this association was largely nullified in their sibling-controlled analysis (HR 0.98, 95% CI 0.93 to 1.04), suggesting confounding by familial factors.^(7)^ Further details can be seen in the supplemental material: table S2.

### Other neurodevelopment findings in the included studies

Despite not being the main objective of this review, all other outcomes were also collected and organized (Supplemental material: Table S3), and prenatal acetaminophen exposure and ADHD diagnosis was noted.^(7,12,15,16,19,20)^ However, familial confounding should be investigated. A strong dose-response relationship between fetal acetaminophen exposure, measured by cord plasma biomarkers, and ADHD diagnoses was observed, increasing the odds of an ADHD diagnosis in 2.25 to 2.86 times.^(19)^ Self-reported maternal acetaminophen use and an increased risk of ADHD symptoms in children (RR 1.45, 95% CI 1.18 to 1.78) was also noted.^(20)^ However, a potential genetic confounding pathway was discussed due to a 1-standard deviation increase in maternal ADHD polygenic risk score was associated with an 11% increase in the odds of acetaminophen use during late pregnancy (OR 1.11, 95% CI 1.04 to 1.18).^(20)^

Beyond overall ADHD diagnoses and symptoms, neurodevelopmental findings involved hyperactivity/impulsivity and attention. Exposed children showed an increased risk of presenting more hyperactivity/impulsivity symptoms (IRR 1.41, 95% CI 1.01 to 1.98), with a significant trend observed for increasing frequency of acetaminophen use.^(17)^ For attention functions, children demonstrated a greater risk of commission errors (IRR 1.10, 95% CI 1.03 to 1.17) and lower detectability scores (coefficient b = −0.07, 95% CI −0.12 to −0.02).^(17)^ Persistent acetaminophen exposure was linked to more K-CPT omission errors (IRR 1.29, 95% CI 1.02 to 1.64).^(17)^ Gender-specific effects on attention were also noted, with persistent exposure in females associated with a higher risk of commission errors (IRR 1.32, 95% CI 1.05 to 1.66) and poorer detectability (coefficient b = −0.18, 95% CI −0.36 to 0.00).^(17)^ However, this study found no statistically significant associations for general mental and psychomotor development (Bayley Scales of Infant Development - BSID), cognitive and motor development (McCarthy Scales of Children's Abilities - MCSA), or social competence (California Preschool Social Competence Scale - CPSCS).^(17)^

Ever-use of acetaminophen during pregnancy was associated with a marginally increased risk of ADHD (HR 1.07, 95% CI 1.05 to 1.10) and intellectual disability (HR 1.05, 95% CI 1.00 to 1.10).^(7)^ However, sibling-controlled analysis found no significant association between acetaminophen use and ADHD (HR 0.98, 95% CI 0.94 to 1.02) or intellectual disability (HR 1.01, 95% CI 0.92 to 1.10), thus indicating substantial confounding by unmeasured familial factors.^(7)^

### Risk of bias in studies

**Figure 4:**
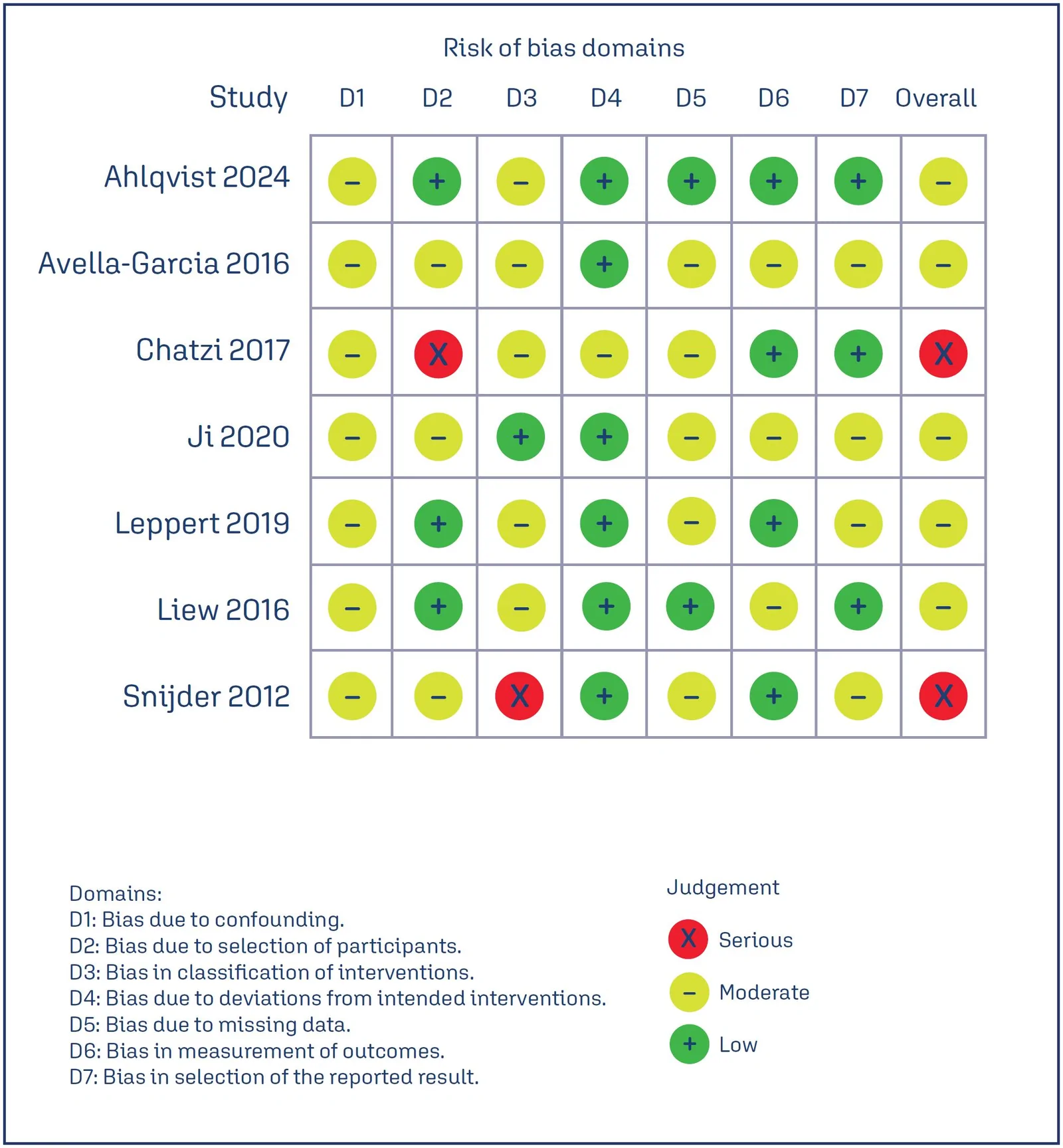
Traffic-light plot for risk of bias assessment of non-randomized studies of exposure

**Figure 5:**
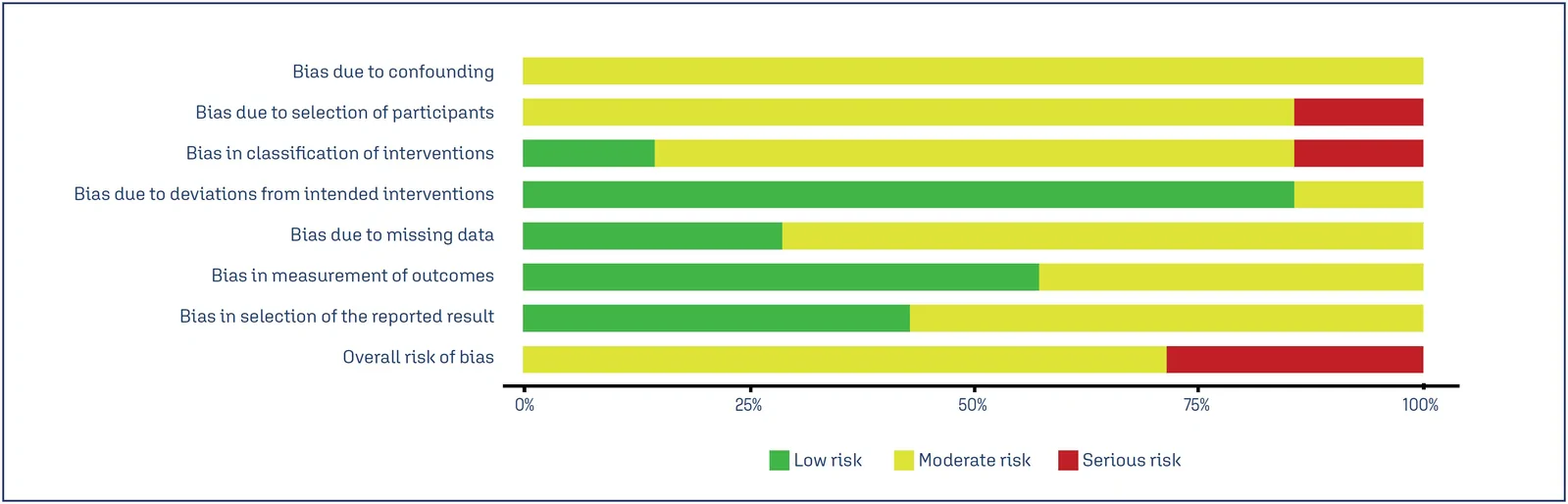
Summary plot for risk of bias assessment of non-randomized studies of exposure

The bias analysis reveals a predominant pattern of "some concerns" in the overall risk of bias,^(5,7,17,18-20)^ with two studies categorized as "high" risk.^(5,16)^ Studies frequently faced "some concerns" in confounding, selection of participants, and classification of exposure, primarily due to the inherent challenges of observational research. For instance, self-reported exposure data often introduced moderate misclassification,^(7,17,18)^ while residual confounding remained a concern despite adjustments. Issues with participant selection and missing data also contributed to "some concerns" risk in several studies, often stemming from differential follow-up or exclusions that could bias the observed associations. The consistent "low" risk for reverse causality across most studies indicates that the temporal sequence of exposure preceding outcome was generally well-established. The two studies with "high" overall risk of bias,^(5,16)^ were driven by: significant issues with differential attrition or selection of patients into the study;^(16)^ and serious concerns in exposure classification, stemming from imprecise self-reported analgesic use without detailed information on dosage or frequency or reassurance of use.^(5)^ The traffic light and summary plot of the risk of bias assessment are respectively in the Figure 4 and 5.

A specific study was included in the analysis due to its results report, despite the non-retrieval of the full paper.^(15)^ However, due to the lack of access to the full paper, risk of bias was unable to be performed. A full paper was requested, with no response. We are open to submit a comment on the meta-analysis in the future, if the full paper is ever retrieved. We also highlight that we performed the leave-one-out analysis which showed the results of the analysis without the study, with no relevant impact on heterogeneity (varied from 15.1% to 25.8% without it) or statistical significance of the findings (no changes on OR and 95% CI).

## Discussion

This systematic review and meta-analysis assessed the association between prenatal acetaminophen use and the risk of ASD in offspring. Our pooled random-effects model, encompassing eight studies and over 2.5 million patients, revealed a statistically significant, albeit modest, increase in the risk of ASD diagnosis, and a non-significant in ASD symptoms.

Acetaminophen, or paracetamol, is known to cross the placental barrier, meaning that maternal exposure directly translates to fetal exposure.^(8,25,26)^ The developing fetal brain is highly vulnerable to external insults, such as medications, prenatal stress and infections, during critical periods of neurodevelopment.^(27)^ Although the precise mechanism involving neurodevelopment is not fully elucidated, several hypotheses exist.^(27)^

Acetaminophen is a prostaglandin synthesis inhibitor,^(28)^ and prostaglandin's role is still being understood, while recognizing its crucial roles in brain development, including neuronal migration, differentiation, and synaptic plasticity.^(29)^ Alterations in these pathways due to prenatal acetaminophen exposure could theoretically disrupt normal neurodevelopmental processes.^(30)^ Moreover, acetaminophen metabolism involves detoxification pathways that can deplete glutathione stores, which is a major antioxidant, essential for protecting cells, including neurons, from oxidative stress. Oxidative stress has been implicated in the pathophysiology of neurodevelopmental disorders like ASD and ADHD and enhances the importance of understanding dose limit during pregnancy, since the maximum intake in adults is of 4g/day and in children of 50 to 75mg per kilo/day.^(31)^ Therefore, maternal or fetal glutathione depletion due to acetaminophen theoretically increases susceptibility to oxidative damage in the developing brain, leading to long-term neurodevelopmental consequences.^(32)^

ASD is characterized by difficulties in social interaction and communication, and restricted or repetitive patterns of behavior, interests, or activities.^(32)^ An ASD diagnosis impacts the quality of life for both the child and their family. Children with ASD frequently require specialized interventions, such as behavioral, speech and occupational therapy and educational support, which can lead to considerable financial and emotional demands.^(33)^

For parents, an ASD diagnosis for their child can be associated with increased stress, anxiety, and even depression.^(34,35)^ The demands of caregiving can affect parents’ employment, social lives, and marital relationships.^(36)^ Consequently, any potential environmental risk factor, even one with a modest effect size, merits careful consideration given its implications for individual well-being and family functioning.

Navigating the evidence regarding acetaminophen use during pregnancy presents a challenge for healthcare professionals and expectant parents. The relief of pain and fever in pregnant women is important, as untreated maternal fever, for example, can pose risks to fetal development, including increased risk of neural tube defects or premature labor.^(37)^ This highlights the immediate benefits of acetaminophen for maternal well-being and fetal health in certain circumstances.

However, the emerging evidence suggesting a link to neurodevelopmental disorders like ASD and ADHD introduces a significant negative factor into this equation. The long-term implications for the child, as discussed previously, are profound. Healthcare providers are now faced with the delicate task of weighing these immediate maternal benefits against potential, albeit still debated and complex, long-term risks for the offspring. This requires a careful, individualized benefit-risk assessment that involves thorough communication with the pregnant woman and her family.^(38)^ Professionals must educate families about the current scientific understanding, acknowledging both the continuing uncertainty and the growing concerns.^(39)^ This conversation should cover the importance of using the lowest effective dose for the shortest possible duration, considering non-pharmacological alternatives where appropriate, and discussing the specific indications for acetaminophen use.^(2)^

As strengths, this systematic review and meta-analysis demonstrates considerable methodological rigor, enhancing the credibility and generalizability of its findings. The comprehensive search strategy across major databases maximized the retrieval of relevant evidence, minimizing selection bias. Furthermore, inclusion of only high-quality randomized controlled trials and cohort studies strengthens the overall evidence base, along with a pooled random-effects model coupled with sensitivity analyses, further confirms the consistency and reliability of the primary findings.

Despite its strengths, this review faces several limitations, largely reflecting the challenges inherent in observational research on prenatal exposures. Key constraints were: (1) Considerable heterogeneity in acetaminophen exposure definitions (dose, duration, or frequency) across studies, ranging from potentially biased self-reported use to more objective; (2) Persistent concerns regarding confounding factors, particularly by underlying maternal conditions (e.g., pain, fever) and unmeasured familial elements; (3) Inconsistency in the ascertainment of neurodevelopmental outcomes, such as varied ages at ASD diagnosis, also introduces heterogeneity; (4) Lack of subgroup analysis due to the relatively small number and data from studies; and (5) Inclusion of observational study only, which can suggest associations but not definitively prove causality due to the persistent risk of residual confounding.

Future research should prioritize addressing the limitations identified in the current body of literature to strengthen causal inference. This includes implementing prospective cohort studies with meticulously collected and standardized data on acetaminophen exposure, encompassing precise details on dose, duration, timing within pregnancy, and the specific indications for its use, ideally supplemented with objective biomarker assessments. Crucially, methodologies to control for confounding factors, particularly unmeasured familial confounders and confounding by indication, are needed. Furthermore, refining the diagnostic ascertainment of neurodevelopmental outcomes, ensuring consistent criteria and long-term follow-up, is vital. Finally, a deeper exploration into the underlying physiological mechanisms by which acetaminophen might influence fetal neurodevelopment is warranted to fully elucidate the pathways leading to altered neurodevelopmental trajectories.

## Conclusion

While acknowledging acetaminophen's historical role as a first-line medication during pregnancy, this review identified a consistent, albeit modest, association between prenatal exposure and an increased risk of autism spectrum disorder diagnosis in the prenatal exposed offspring. Despite the controversies and the complexities introduced by confounding factors and varying methodologies, the aggregated evidence warrants heightened attention due to the high public health stakes of these disorders. The findings underscore the critical need for a careful benefit-risk assessment by healthcare providers, aligning with recent advisories to minimize acetaminophen prescription during pregnancy, and emphasize the importance of ongoing dialogue with expectant mothers to ensure informed decision-making regarding pain and fever management.

## Data Availability

The research data are described in the article presented.
