# Supplementary material for: Prenatal exposure to paracetamol and risk of autism spectrum disorder: systematic review and meta-analysis of observational studies
Source: Rev Bras Ginecol Obstet. 2026 May 12;48:e-rbgo108. doi: 10.61622/rbgo/2026rbgo108 (PMC13399481; doi:10.61622/rbgo/2026rbgo108)
Supplement: Supplemental Material [file 1806-9339-rbgo-48-e-rbgo108-Supp01.pdf]

# Supplementary material

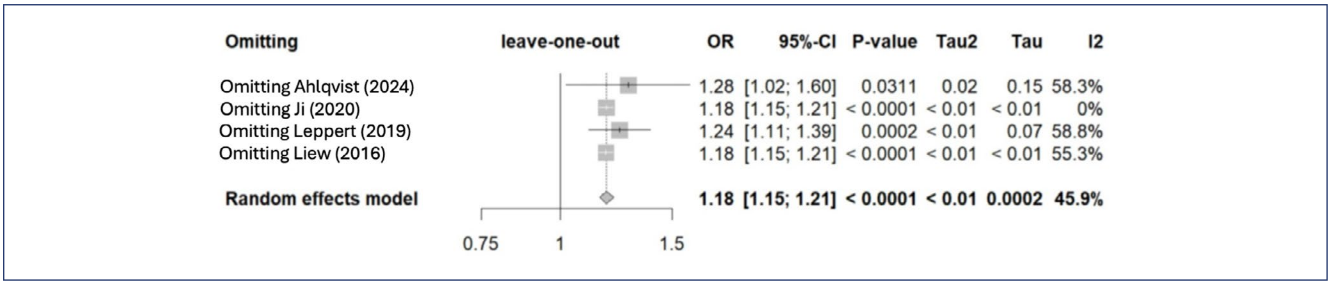

**Figure S1.** Leave-one-out analysis for the outcome of ASD diagnosis

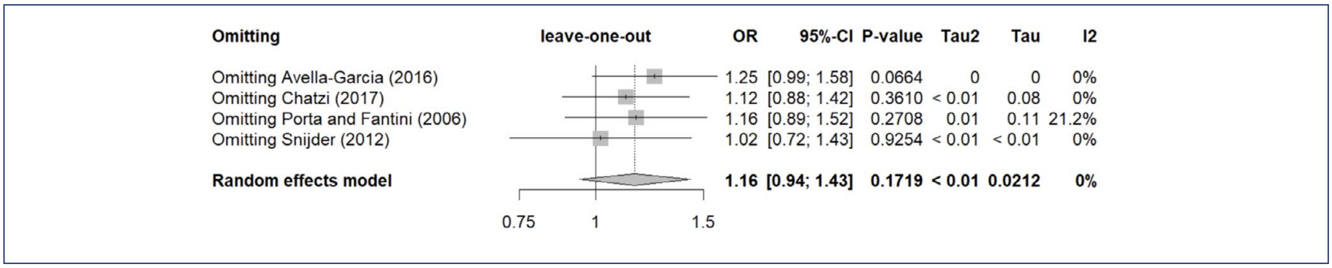

**Figure S2.** Leave-one-out analysis for the outcome of ASD symptoms

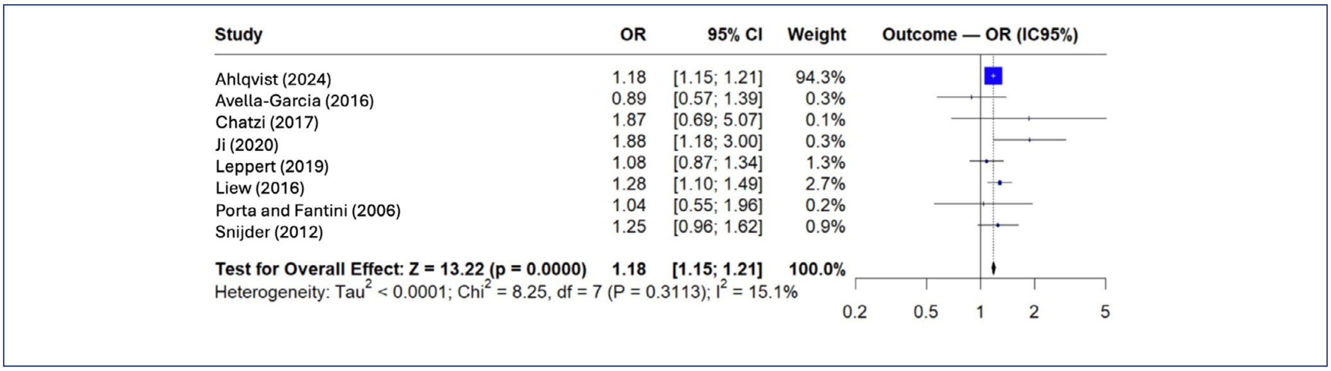

**Figure S3.** Pooled analysis of studies showed higher odds of autism disorder symptoms or diagnosis in the use of acetaminophen during pregnancy when compared with the not-exposed group

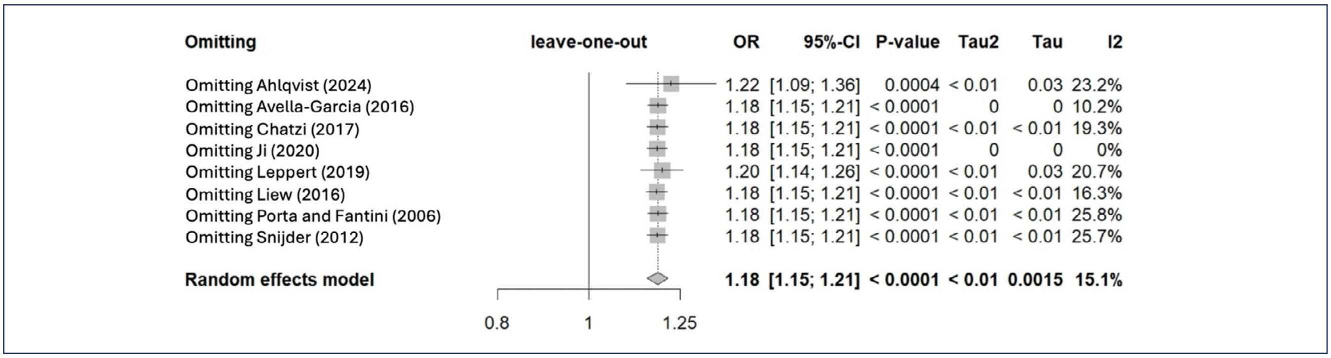

**Figure S4.** Leave-one-out analysis with the omission of individual studies showing low heterogeneity

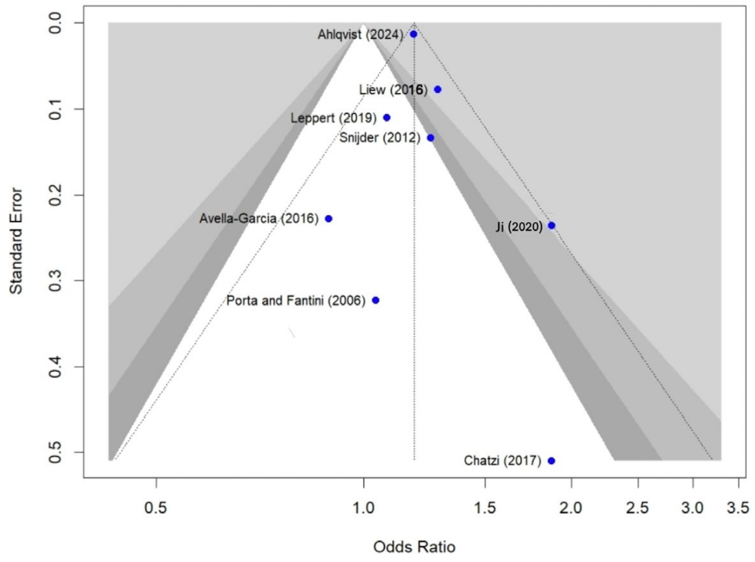


**Figure S5.** Funnel plot for the overall ASD identification (diagnosis and symptoms)

**Table S1.** Search strategy of the included studies

| Database | Search strategy |
|---|---|
| Pubmed | ("N-Acetyl-p-aminophenol" OR Acetamidophenol OR Acetominophen OR Acetaminophen OR APAP OR "p-Acetamidophenol" OR "p-Hydroxyacetanilide" OR Paracetamol OR Hydroxyacetanilide OR "N-(4-Hydroxyphenyl)acetanilide" OR Algotropyl OR Acamol OR "Anacin-3" OR Anacin3 OR Anacin 3 OR Datril OR Panadol OR Tylenol OR Acetaco OR Acephen) AND ("Autism spectrum disorder" OR autistic OR autism OR ASD OR "asperger's syndrome" OR asperger OR "pervasive development disorders" OR PDD OR "childhood disintegrative disorder" OR "child development disorders, pervasive" OR "Pervasive Development Disorders" OR "kanner's syndrome" OR "kanners syndrome" OR "kanner syndrome") |
| Embase and Cochrane | ("N-Acetyl-p-aminophenol" OR Acetamidophenol OR Acetominophen OR Acetaminophen OR APAP OR "p-Acetamidophenol" OR "p-Hydroxyacetanilide" OR Paracetamol OR Hydroxyacetanilide OR "N-(4-Hydroxyphenyl)acetanilide" OR Algotropyl OR Acamol OR "Anacin-3" OR Anacin3 OR Anacin 3 OR Datril OR Panadol OR Tylenol OR Acetaco OR Acephen) AND ("Autism spectrum disorder" OR autistic OR autism OR ASD OR "aspergers syndrome" OR asperger OR "pervasive development disorders" OR PDD OR "childhood disintegrative disorder" OR "child development disorders, pervasive" OR "Pervasive Development Disorders" OR "kanners syndrome" OR "kanner syndrome") |

**Table S2.** Summary of findings associated to autism and hyperkinetic disorder

| Study (Author, Year) | Outcome | Exposure Level/Group | Cases (N) / Total (N) | Adjusted HR/OR (95% CI) |
|---|---|---|---|---|
| Liew (2016) | Autistic spectrum disorders (Overall) | Never used | 401 / 358,257 person-years | 1.00 (ref) |
| | | Ever used | 626 / 460,130 person-years | 1.19 (1.04–1.35) |
| | Infantile autism (Overall) | Never used | 139 / 355,987 person-years | 1.00 (ref) |
| | | Ever used | 206 / 456,439 person-years | 1.10 (0.89–1.37) |
| | Autism spectrum disorders with Hyperkinetic symptoms | Never used (ASD without HK) | 295 cases (295 / cohort, ref for this specific analysis) | 1.00 (ref) |
| | | Ever used (ASD with HK) | 412 cases (412 / cohort) | 1.51 (1.19–1.92) |
| | Infantile autism with Hyperkinetic symptoms | Never used (Inf. Aut. without HK) | 110 cases (110 / cohort) | 1.00 |
| | | Ever used (Inf. Aut. with HK) | 147 cases (147 / cohort) | 1.55 (0.98–2.45) |
| | Asperger syndrome with Hyperkinetic symptoms | Maternal use (with HK) | Not specified in detail | Positively associated (Supporting Info Table S1) |
| | PDD-NOS with Hyperkinetic symptoms | Maternal use (with HK) | Not specified in detail | Positively associated (Supporting Info Table S1) |
| Ji (2020) | ADHD only | Cord unchanged acetaminophen (First tertile) | 62 / 332 | NA |
| | | Cord unchanged acetaminophen (Second tertile) | 75 / 332 | 2.26 (1.40–3.69) |
| | | Cord unchanged acetaminophen (Third tertile) | 120 / 332 | 2.86 (1.77–4.67) |
| | ASD only | Cord unchanged acetaminophen (First tertile) | 12 / 332 | NA |
| | | Cord unchanged acetaminophen (Second tertile) | 21 / 332 | 2.14 (0.93–5.13) |
| | | Cord unchanged acetaminophen (Third tertile) | 33 / 332 | 3.62 (1.62–8.60) |
| Ahlqvist et al. (2024) | Autism (Population-based model) | Ever-use vs. No use | 5857 exposed cases / 185,909 | HR 1.05 (1.02–1.08) |
| | | Risk Difference at 10 years | NA | RD 0.09% (−0.01% to 0.20%) |
| | Autism (Sibling control model) | Ever-use vs. No use | 3956 exposed cases / 1,773,747 (sibling cohort) | HR 0.98 (0.93–1.04) |
| | | Risk Difference at 10 years | NA | RD 0.02% (−0.14% to 0.18%) |
| | | Low dose (<166 mg/d) vs. No use | NA | HR 0.85 (0.68–1.08) |
| | | High dose (≥430 mg/d) vs. No use | NA | HR 0.88 (0.68–1.14) |

Continue...

Continuation.

| | | | | |
|---|---|---|---|---|
| Avella-Garcia et al. (2016) | CAST all children (ever-exposed vs. non-exposed) | CAST males (ever-exposed) | 751 (total exposed males with data) | b 0.63 (0.09–1.18) |
| | | CAST females (ever-exposed) | 716 (total exposed females with data) | b −0.51 (−0.98–−0.05) |
| | CAST males (frequency of use) | Sporadic use vs. Never use | 272 (sporadic exposed males) | b 0.56 (0.001–1.11) |
| | | Persistent use vs. Never use | 21 (persistent exposed males) | b 1.91 (0.44–3.38) |
| Chatzi 2017 (RHEA Cohort from Alemany 2021) | Autistic Spectrum Symptoms (CBCL 6/18) | Prenatal acetaminophen exposure | 345 (cohort size) | OR ~1.06 (0.77–1.45) (Visual est. from Alemany Fig. 1A) |
| Porta 2006 (GASPII Cohort from Alemany 2021) | Autistic Spectrum Symptoms (PDP subscale of CBCL1½–5) | Prenatal acetaminophen exposure | 153 (cohort size) | OR ~1.15 (0.81–1.62) (Visual est. from Alemany Fig. 1A) |

Note: For Liew et al. (2015), the 'Total (N)' for 'Cases (N) / Total (N)' refers to person-years for overall ASD/Infantile Autism, and case counts within the entire cohort for hyperkinetic subtypes where specific denominators for exposed/unexposed for that specific outcome were not provided. For Ji et al. (2019), the 'Total (N)' represents the number of participants within that specific tertile or detection group for the cord biomarker. For Ahlqvist et al. (2024), "Cases (N) / Total Exposed (N)" reflects the number of observed neurodevelopmental cases within the exposed group (or sibling cohort for sibling analysis) as given in Figure 3 of the paper, not the total number of individuals in the exposed group. The primary finding from their sibling analysis is the adjusted HR. For Avella-Garcia et al. (2016), "Total Exposed (N)" refers to the 'Nc' value, which is the number of subjects with acetaminophen exposure, neurodevelopment outcome, and potential confounders available for that specific analysis. 'NA' denotes not applicable or not directly reported as a specific count in the original paper for that specific result type. For Chatzi 2017 (RHEA Cohort) and Porta 2006 (GASPII Cohort), the reported ORs are visual estimations from the forest plots in Figure 1A of Alemany et al. (2021) meta-analysis.

**Table S3.** Summary of findings of other neurodevelopmental and related outcomes.

| Study (Author, Year) | Outcome | Exposure Level/Group | Cases (N) / Total (N) | Adjusted HR/OR (95% CI) |
|---|---|---|---|---|
| Leppert 2019 | ADHD symptoms (observational association) | Acetaminophen use during pregnancy | Not specified / 7486 (N for observational analysis) | RR 1.45 (1.18–1.78) |
| | Maternal ADHD PRS associated with acetaminophen use in late pregnancy | 1-SD increase in ADHD PRS | Not specified / 7921 (mothers) | OR 1.11 (1.04–1.18) |
| Snijder 2012 | Cryptorchidism | No mild analgesic use during 14-22 weeks gestation | Not specified / cohort of 3184 boys | 1.00 (ref) |
| | | Mild analgesic use during 14-22 weeks gestation | 17 / 480 (16.8% of 2864 women reported use) | 2.12 (1.17–3.83) |
| | Cryptorchidism | No paracetamol use during 14-22 weeks gestation | Not specified / cohort of 3184 boys | 1.00 (ref) |
| | | Paracetamol use during 14-22 weeks gestation | 15 / 448 (16.1% of 2864 women reported use) | 1.89 (1.01–3.51) |
| | Hypospadias | Mild analgesic use (any period) | 7 / 22 cases with hypospadias | No significant association (due to small case number) |
| Ahlqvist et al. (2024) | ADHD (Population-based model) | Ever-use vs. No use | 12714 exposed cases / 185,909 | HR 1.07 (1.05–1.10) |
| | | Risk Difference at 10 years | NA | RD 0.21% (0.08%–0.34%) |
| | ADHD (Sibling control model) | Ever-use vs. No use | 8526 exposed cases / 1,773,747 | HR 0.98 (0.94–1.02) |
| | | Risk Difference at 10 years | NA | RD −0.02% (−0.21% to 0.15%) |
| | Intellectual Disability (Sibling control model) | Ever-use vs. No use | 1350 exposed cases / 1,773,747 | HR 1.01 (0.92–1.10) |
| | | Risk Difference at 10 years | NA | RD 0.00% (−0.10% to 0.13%) |
| Avella-Garcia et al. (2016) | Hyperactivity/Impulsivity symptoms (ADHD-DSM-IV) | Ever-exposed vs. Non-exposed | 1382 (exposed with data) | IRR 1.41 (1.01–1.98) |
| | K-CPT commission errors | Ever-exposed vs. Non-exposed | 1255 (exposed with data) | IRR 1.10 (1.03–1.17) |
| | K-CPT detectability | Ever-exposed vs. Non-exposed | 1255 (exposed with data) | b −0.07 (−0.12–−0.02) |
| | K-CPT omission errors | Persistent use vs. Never use | 48 (persistent exposed with data) | IRR 1.29 (1.02–1.64) |
| | K-CPT commission errors (females) | Persistent use vs. Never use | 27 (persistent exposed females) | IRR 1.32 (1.05–1.66) |
| | K-CPT detectability (females) | Persistent use vs. Never use | 27 (persistent exposed females) | b −0.18 (−0.36–0.00) |
| | BSID (Mental and Psychomotor Development) at 1 year | Ever-exposed vs. Non-exposed | 955 (exposed with data) | b 0.75 (−0.75–2.25) |
| | MCSA (Cognitive and Motor Development) at 5 years | Ever-exposed vs. Non-exposed | 828 (exposed with data) | b −0.21 (−1.70–1.28) |
| | CPSCS (Social Competence) at 5 years | Ever-exposed vs. Non-exposed | 828 (exposed with data) | b −1.15 (−3.16–0.87) |
| Chatzi 2017 (RHEA Cohort from Alemany 2021) | ADHD Symptoms (CBCL 6/18) | Prenatal acetaminophen exposure | 345 (cohort size) | OR ~1.15 (0.82–1.62) (Visual est. from Alemany Fig. 1B) |
| Porta 2006 (GASPII Cohort from Alemany 2021) | ADHD Symptoms (CBCL1½–5) | Prenatal acetaminophen exposure | 153 (cohort size) | OR ~1.15 (0.79–1.69) (Visual est. from Alemany Fig. 1B) |

Note: For Leppert et al. (2019) observational findings, specific case numbers for exposed/unexposed groups were not provided, only the relative risk for the outcome. For Snijder et al. (2012), the 'Total (N)' for exposure groups represents the number of cases of cryptorchidism found among mothers who reported using mild analgesics during the specified period. For Ahlqvist et al. (2024), "Cases (N) / Total Exposed (N)" reflects the number of observed neurodevelopmental cases within the exposed group (or sibling cohort for sibling analysis), not the total number of individuals in the exposed group. The primary finding from their sibling analysis is the adjusted HR. For Avella-Garcia et al. (2016), "Total Exposed (N)" refers to the 'Nc' value, which is the number of subjects with acetaminophen exposure, neurodevelopment outcome, and potential confounders available for that specific analysis. 'NA' denotes not applicable or not directly reported as a specific count in the original paper for that specific result type. For Chatzi 2017 (RHEA Cohort) and Porta 2006 (GASPII Cohort), the reported ORs are visual estimations from the forest plots in Figure 1B of Alemany et al. (2021) meta-analysis.
